# Evaluation of Shape Recovery Performance of Shape Memory Polymers with Carbon-Based Fillers

**DOI:** 10.3390/polym16172425

**Published:** 2024-08-27

**Authors:** Sungwoong Choi, Seongeun Jang, Seung Hwa Yoo, Gyo Woo Lee, Duyoung Choi

**Affiliations:** 1 Carbon & Light Material Application Research Group, Korea Institute of Industrial Technology, Jeonju 54853, Republic of Korea; 2 Division of Mechanical Design Engineering, Jeonbuk National University, Jeonju 54896, Republic of Korea; 3 Division of Chemical Engineering, Jeonbuk National University, Jeonju 54896, Republic of Korea; 4 Department of Quantum System Engineering, Jeonbuk National University, Jeonju 54896, Republic of Korea; 5 Department of JBNU-KIST Industry-Academia Convergence Research, Graduate School, Jeonbuk National University, Jeonju 54896, Republic of Korea; 6 Convergence Research Center for Recyclable Air Mobility, Materials and Platform, Korea Institute of Science and Technology, Wanju-gun 55324, Republic of Korea

**Keywords:** shape memory polymer composites, shape memory performance, shape memory effect, composites, carbon fiber, graphite, epoxy, mixed filler

## Abstract

This study focuses on enhancing the thermal properties and shape recovery performance of shape memory polymers (SMPs) through the application of carbon-based fillers. Single and mixed fillers were used to investigate their effects on the glass transition temperature (T_g_), thermal conductivity, and shape recovery performance. The interaction among the three-dimensional (3D) structures of mixed fillers played a crucial role in enhancing the properties of the SMP. These interactions facilitated efficient heat transfer pathways and conserved strain energy. The application of mixed fillers resulted in substantial improvements, demonstrating a remarkable 290.37% increase in thermal conductivity for SMPCs containing 60 μm carbon fiber (CF) 10 wt% + graphite 20 wt% and a 60.99% reduction in shape recovery time for SMPCs containing CF 2.5 wt% + graphite 2.5 wt%. At a content of 15 wt%, a higher graphite content compared to CF improved the thermal conductivity by 37.42% and reduced the shape recovery time by 6.98%. The findings demonstrate that the application of mixed fillers, especially those with high graphite content, is effective in improving the thermal properties and shape recovery performance of SMPs. By using mixed fillers with high graphite content, the performance of the SMP showed significant improvement in situations where fast response times were required.

## 1. Introduction

Shape memory polymer (SMP) is a smart material with the shape memory effect (SME), enabling it to return from a deformation shape to its original shape when exposed to various stimuli such as moisture [1], light [2], electricity [3], and heat [4]. Compared to shape memory alloy (SMA) with the same SME, SMP offers the advantages of lower cost, lower density, excellent formability, and superior recovery performance, making it suitable for applications in electronics, fiber, medical devices, and more [5,6]. However, one disadvantage of SMP activated by heat is its low thermal conductivity due to its organic nature [7]. Previous studies have reported a thermal conductivity of 0.2296 W/mK for SMP, confirming its inherently poor heat conduction capability [8]. Low thermal conductivity negatively impacts shape recovery time, making it difficult to apply SMP to applications that require fast shape recovery, such as biomedical materials or sensors.

SMP is influenced by the ratio of epoxy resin to curing agent, affecting its glass transition temperature (T_g_) and thermal, electrical, and mechanical properties [9,10]. However, not all epoxies exhibit SME; only those that have been formulated with specific curing agents and conditions demonstrate this effect. The formulation used in this study is designed to exhibit SME by optimizing the ratio of epoxy resin to hardener, allowing for the necessary storage and release of strain energy during thermal cycling. T_g_ is a significant factor in SMP because it determines the cure temperature, time, and operating temperature for shape recovery. Specifically, SMP can be deformed into the desired shape above T_g_, while below T_g_, the shape is fixed due to the restricted movement of the polymer chains. The stored strain energy is released as the polymer chains move, causing an entropy change and allowing the SMP to recover its original shape [11]. Previous studies have investigated the effect of the epoxy resin to curing agent ratio on T_g_ and shape recovery performance, aiming to identify the optimal ratio [8].

As mentioned earlier, SMP has low thermal conductivity due to its organic nature. There is active research on using thermally conductive fillers in SMP to increase thermal conductivity [12,13]. One way to improve the thermal conductivity of SMP is to add carbon-based fillers such as carbon fiber (CF), graphite, and carbon nanotubes (CNTs). CF has a thermal conductivity of 100 W/mK, while graphite has a thermal conductivity of 1950 W/mK in the plane direction and 5 W/mK in the vertical direction, making it an effective filler for improving thermal conductivity. Several studies have demonstrated the successful use of CF to increase thermal conductivity [14,15]. Moreover, graphite has shown promising results in improving thermal conductivity [16,17].

There is also active research on improving the thermal conductivity of SMP not only by using fillers as a single filler but also by mixing fillers of different types, shapes, and sizes [18,19]. In particular, mixing fillers with different geometries can create unique structures, forming a three-dimensional (3D) mixture of one-dimensional (1D) and two-dimensional (2D) structures. The 3D structure facilitates the formation of thermal paths between fillers within the composites, which contributes to improved thermal properties [20]. Additionally, it increases the density of the material, which has a positive effect on its mechanical properties. In this case, in a mixture with a 3D filler structure, the filler mix ratio plays an important role in determining the thermal conductivity, mechanical properties, and shape recovery performance of the SMP [21].

In this study, shape memory polymer composites (SMPCs) were prepared by using CF and graphite as single and mixed fillers in SMP. The effect of varying the ratio and content of CF and graphite on SMPCs was then compared. The prepared SMPC samples were evaluated for T_g_, thermal conductivity, and shape recovery performance. Single and mixed fillers were used at 5, 10, 15, 20, and 30 wt%, and the 15 wt% mixed filler was compared with different ratios of graphite to CF. The purpose of this research is to determine the optimal filler type, ratio, and content in an SMP that can improve thermal conductivity and shape recovery performance. By enhancing the properties of SMP, this research highlights potential applications in areas such as biomedical devices and sensors where fast shape recovery time is important.

## 2. Materials and Methods

### 2.1. Materials

**Figure 1:**
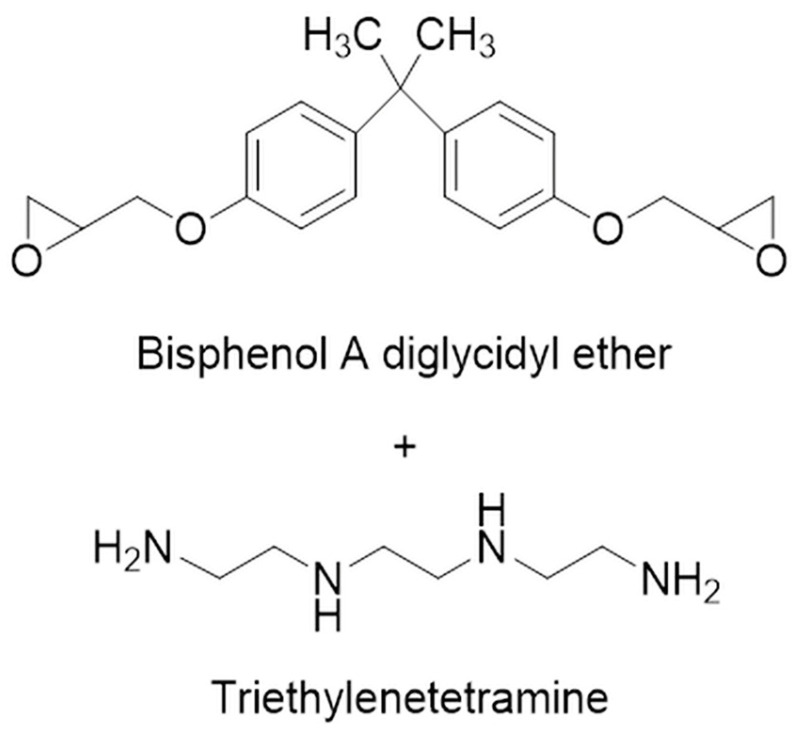
Chemical structure of SMP.

**Figure 2:**
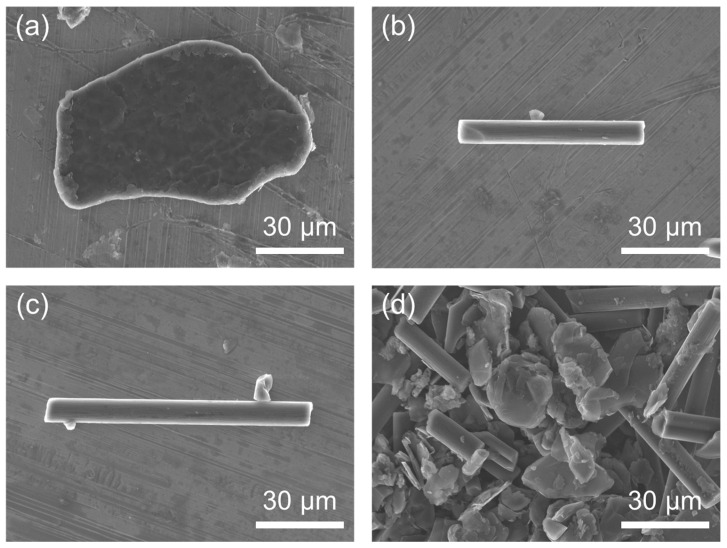
FE-SEM image of the fillers: (**a**) graphite; (**b**) 60 μm CF; (**c**) 100 μm CF; (**d**) mixed filler.

**Figure 3:**
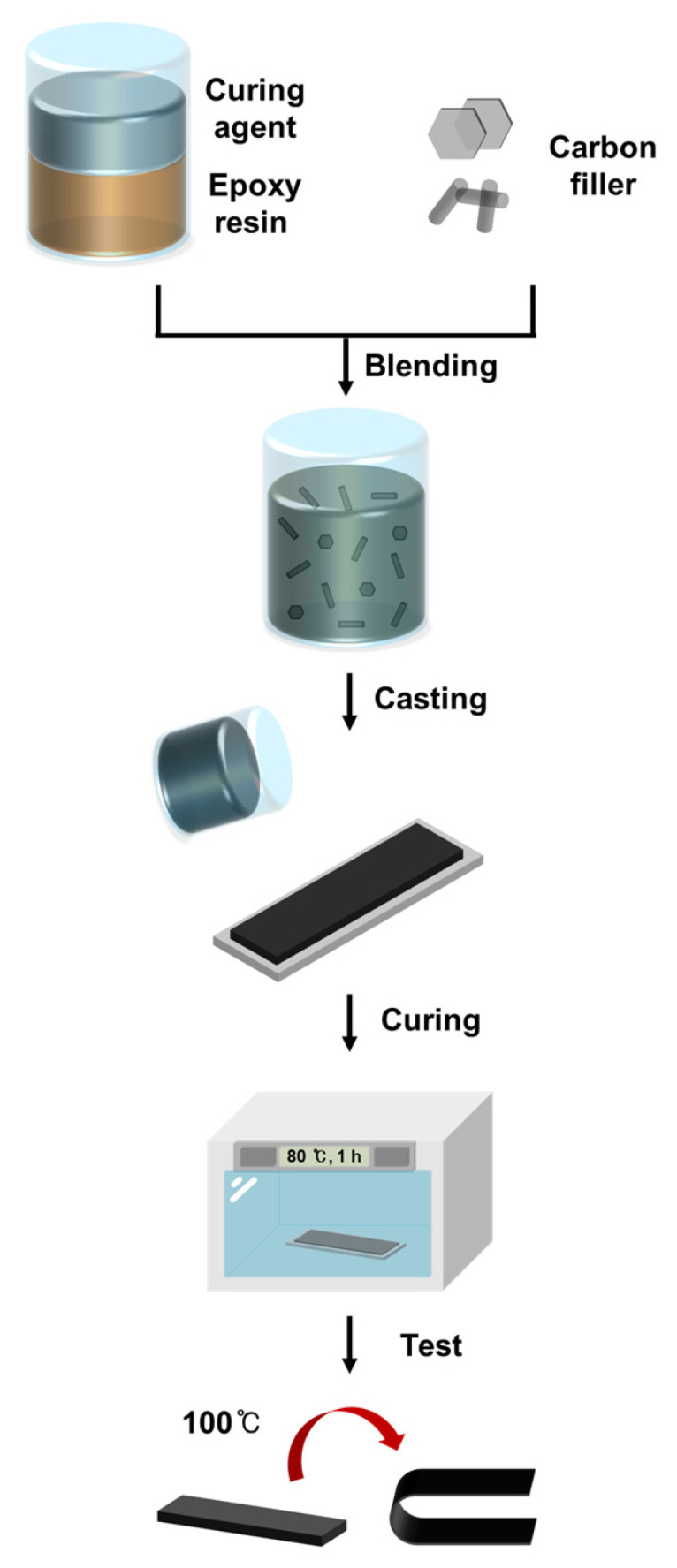
Schematic diagram of SMP specimen preparation process.

The SMP was prepared by mixing a bisphenol-A epoxy resin (density 1.11 g/cm^3^) with the hardener triethylenetetramine (density 0.97 g/cm^3^) in a weight ratio of 10:1, which was determined based on previous research [8]. Figure 1 shows the chemical structure of the epoxy resin and hardener used to make the SMP. The fillers for the SMP were graphite with a diameter of 100 to 300 μm and CF in powder form with a length of 60 to 100 μm, purchased from Sigma Aldrich (St Louis, MO, USA) and Fiberman (Goyang, Republic of Korea), respectively. Both graphite and CF were used without further treatment. Figure 2 shows FE-SEM images of the filler used for the SMP, where Figure 2a shows graphite with a diameter of approximately 100 to 300 μm, and Figure 2b,c show the CF with lengths of 60 and 100 μm, respectively. Figure 2d shows an image of the filler mixed with graphite and CF. Figure 3 shows an overview of the SMP and SMPC specimen production process, and Table 1 and Table 2 show the weight percentages of the single and mixed fillers used in the SMP. The no-filler sample is identified as neat SMP without filler in the figures. For SMP, the epoxy resin and hardener were mixed in a 10:1 ratio, and the fillers were mixed in the amounts shown in Table 1 and Table 2. The mixture was then dispersed using an ultrasonic disperser for 1 h. After dispersion, the prepared mixture was poured into the mold and cured. The specimens were then cured in an oven set at 80 °C for 1 h. The cured specimens were subjected to thermal conductivity testing, followed by shape recovery testing in an oven set at 100 °C.

### 2.2. Methods

**Figure 4:**
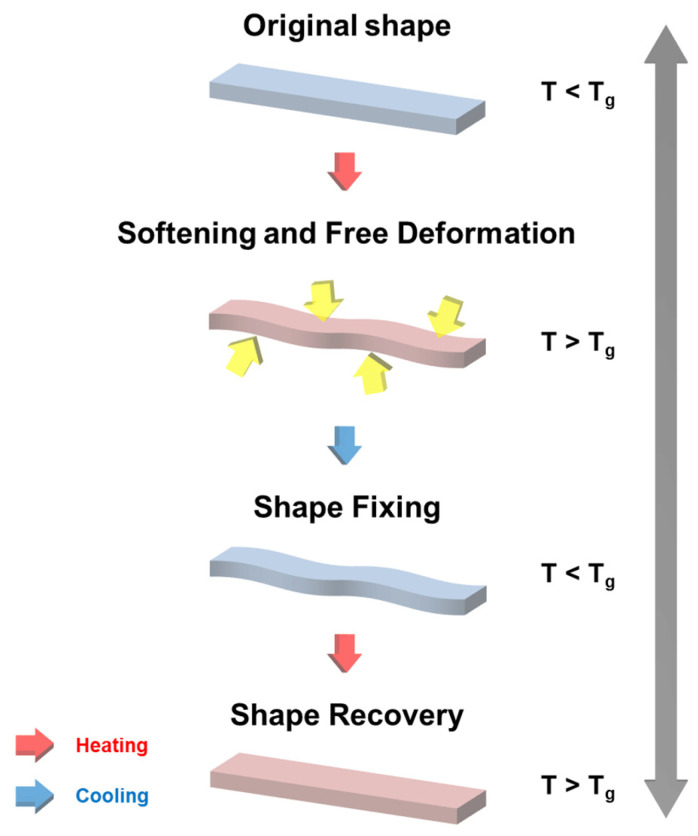
Schematic diagram of the process for testing the shape recovery performance.

**Figure 5:**
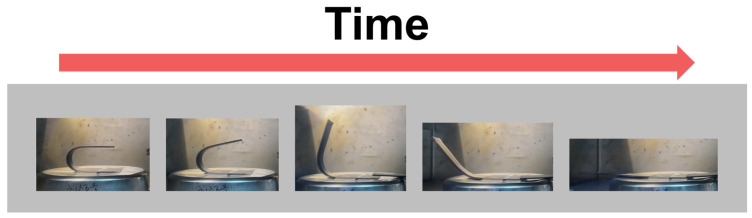
Sequential images of SMP shape recovery from fixed to original shape.

A field emission scanning electron microscope (FE-SEM), model JSM-7100F, manufactured by JEOL, Peabody, MA, USA, was used to evaluate the morphological properties of graphite and CF. The T_g_ of SMP and SMPC was measured by differential scanning calorimetry (DSC), with samples weighing 5 to 10 mg and evaluated using a DSC analyzer (DSC 350, TA Instruments, New Castle, DE, USA) with a temperature range from 30 to 180 °C at a heating rate of 10 °C/min in an N_2_ atmosphere. The thermal conductivity of SMP and SMPC was measured using a thermal conductivity tester (TPS 2500S, Hot Disk, Gothenburg, Sweden). The specimens, each with a diameter of 30 mm and a height of 5 mm, were carefully prepared by polishing the surface with fine-grit sandpaper to ensure a smooth and uniform surface. The measurement was performed by placing a probe between two cylindrical specimens, ensuring intimate contact between the probe and the specimen surfaces. The TPS 2500S uses a transient plane source method, where a sensor simultaneously serves as a heat source and a temperature monitor. The thermal conductivity was measured over a period of 10 s, with each sample being tested five times to ensure accuracy and repeatability. The average value was taken as the final thermal conductivity measurement for each sample. To evaluate the shape recovery performance of SMP and SMPC, we measured the shape fixation ratio, shape recovery ratio, and shape recovery time, respectively. Figure 4 shows the shape recovery process of SMP as T_g_ changes. First, the specimen was heated in an oven at 100 °C for 120 s and then subjected to an external force (indicated by the yellow arrows) to hold it in the U-shaped mold. The fixed specimen was subsequently cooled to room temperature (25 °C) for 300 s, during which the fixed angle (*θ_fixed_*) was measured. The specimen was then reheated to 100 °C to recover its original shape, at which point the bending angle (*θ_i_*) was measured. The maximum angle (*θ_max_*) was recorded when the SMP was fully deformed under the applied force. These measurements *θ_max_*, *θ_fixed_*, and *θ_i_* were used to calculate the shape fixation and shape recovery ratios, ensuring a clear understanding of the results presented in this study. The grey arrows in Figure 4 indicate that the entire procedure of shape fixation and recovery in SMP can be back and forth repeatedly, highlighting the material’s reusability. To further illustrate the shape recovery performance, Figure 5 presents sequential images of the SMP samples transitioning from a fixed shape back to their original shape over time. This visual demonstration complements the quantitative data, providing a clear representation of how the SMP recovers its shape under the experimental conditions described. The shape fixation ratio and shape recovery ratio are shown as percentages:

(1) $$Shape\;fixation\;ratio:\frac{\theta_{fixed}}{\theta_{max}}\times 100\;(\%)$$

(2) $$Shape\;recovery\;ratio\;:\frac{\theta_{max}-\theta_{i}}{\theta_{max}}\times 100\;(\%)$$

**Figure 6:**
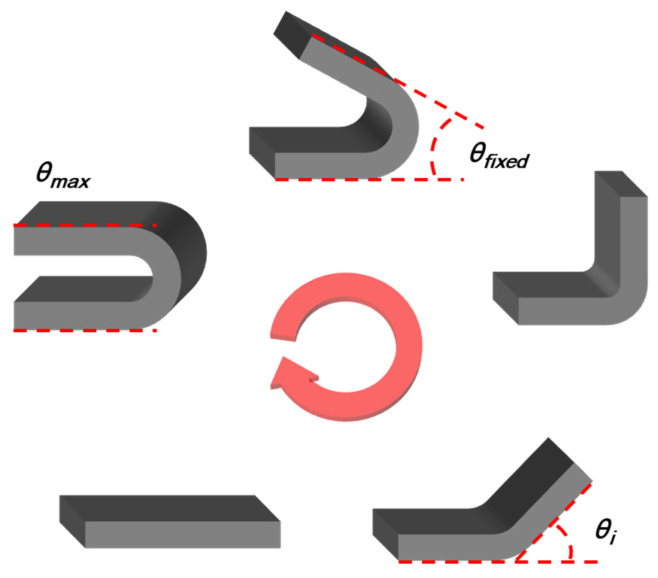
Schematic of SMP shape recovery performance measurement.

Figure 6 shows the *θ_max_*, *θ_fixed_*, and *θ_i_* used to evaluate shape recovery performance. The red arrow in the figure indicates that this process can be repeated, emphasizing the reusability of the shape memory polymer.

## 3. Results and Discussion

### 3.1. Differential Scanning Calorimetry (DSC)

**Figure 7:**
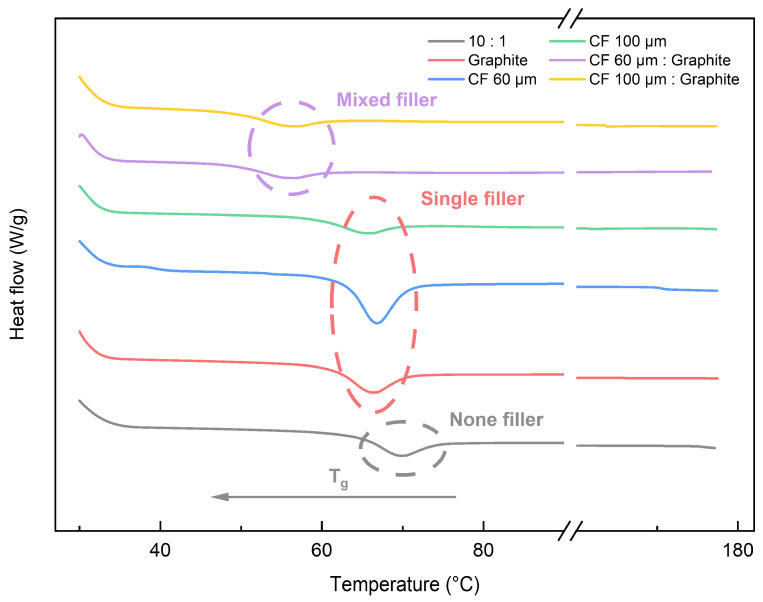
DSC results of SMPC according to the filler.

T_g_ is significant in SMP because it indicates the temperature at which the material transitions from a glassy to a rubbery state, which is critical for shape memory actuation. When the movement of the polymer segments is limited below T_g_, they are considered to be in a glassy state. In general, below T_g_, the shape is fixed, and above T_g_, external forces cause shape deformation. Table 3 shows the change in T_g_ depending on the curing agent ratio. At ratios of 8:1, 9:1, and 10:1, the T_g_ was 83.60, 75.77, and 69.41 °C, respectively. An increase in hardener content results in an increase in T_g_. The variation of T_g_ according to the hardener content shows a similar tendency to the results of previous research [8]. Because the temperature of shape recovery to the original shape is over 100 °C for our SMP, the lower the T_g_, the greater the ∆T between operating temperature and T_g_, which improves shape recovery speed. Thus, when comparing the 8:1 and 10:1 ratios in the previous study, the 10:1 ratio showed better shape recovery performance. In this study, a 10:1 ratio was set as the optimal ratio of epoxy resin and hardener in SMP. Figure 7 shows the DSC results for the SMP with 3 wt% of single and mixed fillers. The dashed circles in the figure highlight the T_g_ transitions for different filler types which the purple, the red and the grey circle indicate the mixed, single and none filler respectively. The application of a single filler lowered the T_g_ of the SMP, and the mixed filler showed a lower T_g_ compared to the single filler, which could be attributed to the 3D structure of the mixed filler. The 3D structure decreases T_g_ because it forms an improved heat transfer path in the polymer matrix [22]. In addition, the interfacial properties between the filler and the SMP matrix help to reduce T_g_ by promoting molecular mobility [22]. We measured T_g_ by using single and mixed fillers through DSC. We found that mixed fillers forming a 3D structure effectively reduced T_g_ compared to single fillers.

### 3.2. Thermal Conductivity

**Figure 8:**
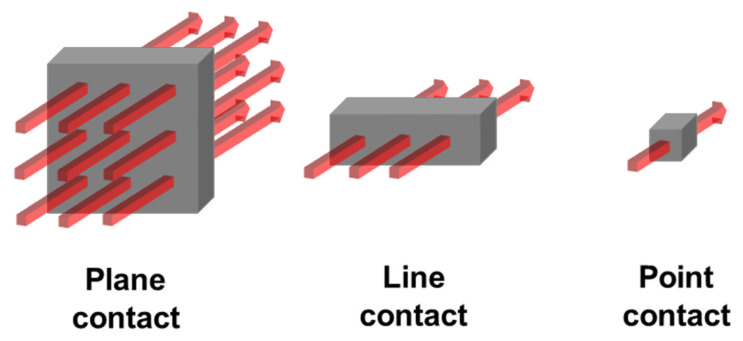
Schematic diagram of the heat transfer path by plane, line, and point.

The thermal conductivity of SMP is determined by the type, content, and shape of the fillers. Low content fillers and insufficient contact points between fillers lead to phonon scattering in the polymer matrix. Hence, the filler composition and content play a crucial role in influencing both interfacial thermal resistance (ITR) and thermal conductivity. Mixed fillers can form 3D structures by mixing fillers with various structures such as 0D, 1D, and 2D. Three-dimensional structures can be achieved through the combination of various dimensional components, such as 0D + 1D or 1D + 2D structures. The 3D structure is advantageous for forming dense networks within the polymer matrix. These structures offer benefits for creating pathways that facilitate heat transfer [22]. The structures formed show point, line, and plane contacts. The large contact area of the planar contact provides a significant advantage in heat transfer because it prevents phonon scattering and is effective in reducing the ITR phenomenon. However, it is important to note that carbon-based nanoparticles, such as CNTs, tend to form agglomerates rather than remaining uniformly dispersed, which can significantly affect their dispersion characteristics and, consequently, the thermal conductivity. Recent studies have shown that the geometry of nanoparticles, such as their length and diameter, plays a crucial role in their dispersion within the polymer matrix [23,24]. As the filler size decreases, the contact area between fillers increases, potentially enhancing heat transfer efficiency, but agglomeration can disrupt this effect by increasing ITR. Figure 8 schematically shows heat transfer pathways for planes, lines, and points. The red arrows in the figure indicate the relative amount of heat transfer through these pathways, illustrating how the structure of the filler (1D, 2D, 3D) affects the efficiency of heat conduction. Specifically, 3D structures with more extensive planar contacts facilitate greater heat transfer compared to 1D or 2D structures.

**Figure 9:**
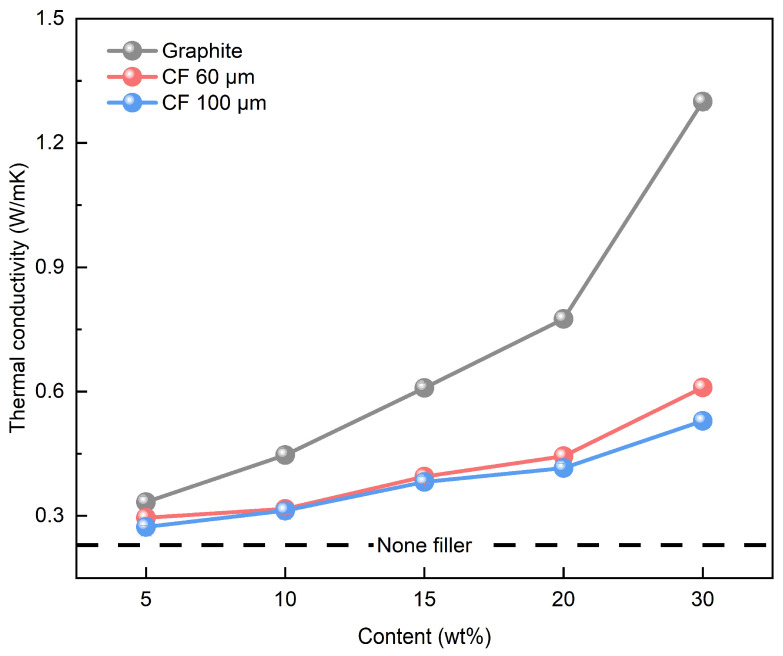
Thermal conductivity results of single filler.

The thermal conductivity of SMP was measured to be 0.2296 W/mK. CFs of lengths 60 and 100 μm were used along with graphite to increase thermal conductivity. The weight ratio of the single filler used is shown in Table 1. Figure 9 shows the thermal conductivity results of the SMP containing single fillers. The increase in filler content creates a large amount of filler-to-filler contact points, increasing thermal conductivity. The critical volume fraction of fillers within the polymer matrix also increased, affecting the formation of heat transfer paths [25,26]. The thermal conductivity of graphite with plane contact was higher than that of CF with line contact. In particular, the thermal conductivity increased to 1.2997 W/mK with 30 wt% graphite, a 466.07% increase. The shorter-length CFs are known to disperse and contact evenly within the polymer matrix, providing better heat transfer path formation [27]. Short CFs are tightly packed together within the polymer matrix to form dense networks. Therefore, the 60 μm CFs were concluded to have higher thermal conductivity than the 100 μm CFs. The gap between thermal conductivities of CF and graphite fillers grows larger after the content of 10 wt%. As shown in Figure 8, the graphite fillers form plane contacts, which offer larger heat transfer paths compared to point contacts. Additionally, an increase in filler content in the polymer matrix further enhances the formation of heat transfer pathways. A higher number of these pathways contributes to an increase in the thermal conductivity of SMPCs.

**Figure 10:**
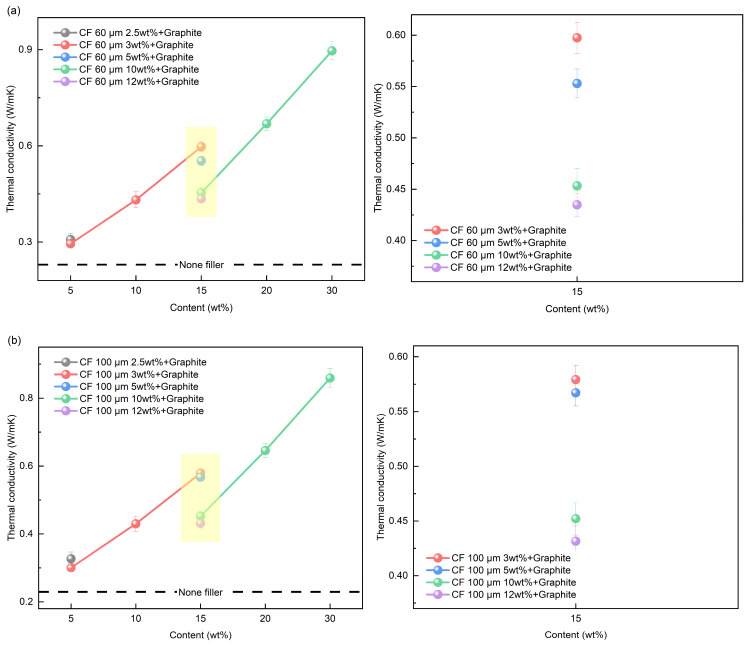
Thermal conductivity results of mixed filler: (**a**) 60 μm CF; (**b**) 100 μm CF.

Previously, we mentioned that mixed fillers are effective at improving thermal conductivity due to their 3D structure. To investigate the effect of mixed filler composition on thermal conductivity, SMPC samples were prepared according to the contents in Table 2. For the 15 wt% mixed filler, different ratios of graphite and CF were used (3:12, 5:10, 10:5, 12:3). Figure 10 shows the thermal conductivity results of the SMP with mixed fillers, where Figure 10a,b show the results with 60 and 100 μm CFs, respectively. Similar to the previous results, the thermal conductivity increased as the mixed filler content increased. It is thought that the increase in the volume fraction of the filler within the polymer matrix is the result of the formation of efficient heat transfer paths. In the 15 wt% samples, different contents of graphite and CF were used. The higher content of graphite resulted in higher thermal conductivity. In particular, the sharp difference in thermal conductivity between 3 and 5 wt% and 10 and 12 wt% can be observed. As depicted in Figure 8, heat is transferred through plane contacts of graphite. An increase in the filler content within the polymer matrix leads to an enhancement of thermal conductivity. Thus, as shown in Figure 9, it appears that higher graphite content correlates with increased thermal conductivity. As a result, the use of mixed fillers with high graphite content enhanced the thermal conductivity of the SMP. Figure 10 shows the thermal conductivities according to CF length.

The 60 μm CF mixed with graphite had higher thermal conductivity than the 100 μm CF and graphite mixed fillers. It indicates that the short CFs and graphite might have formed more contacts because of the steric hindrance of long CFs. Graphite forms mainly plane contacts in the polymer matrix. However, mixed fillers have fewer contact points than the graphite fillers forming point, line, and plane contacts, as shown in Figure 8. The insufficient contacts between the fillers cause phonon scattering in the polymer matrix, which leads to ITR. Therefore, the thermal conductivity of SMPCs with graphite is considered to be superior.

### 3.3. Shape Recovery Performance

#### 3.3.1. Shape Fixation Ratio

**Figure 11:**
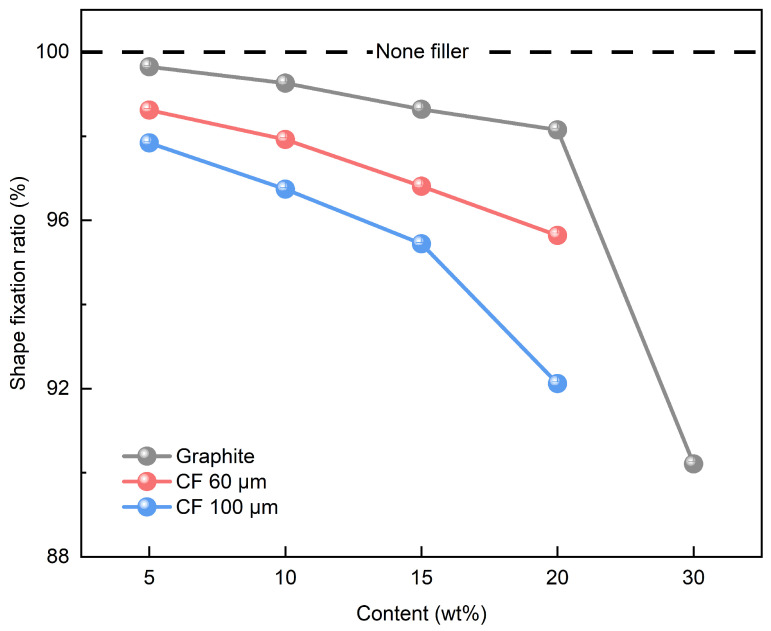
Shape fixation ratio results of single filler.

Figure 4 shows the process of shape fixation in SMP. The process is achieved by restricting the movement of the polymer chains. The shape fixation ratio of the SMP without filler was measured to 100%. The shape fixation ratio of SMP with a single filler was analyzed using the contents in Table 1. Figure 11 shows the shape fixation ratio of SMP with a single filler. The shape fixation ratio decreased with increasing filler content. Increasing filler content within the polymer matrix increases the van der Waals forces between the filler particles and negatively affects the shape fixation ratio [25,26]. The shape fixation ratio of graphite was higher than that of CF. This phenomenon is attributed to the 2D structure of graphite, which better constrains the movement of the polymer chains. Furthermore, when the filler content was 30 wt%, excluding graphite, the specimen fractured during the shape fixation process. Specifically, for 60 and 100 μm CFs, the high filler content resulted in increased brittleness and reduced flexibility of the SMPC, leading to sample fracture during testing. This issue prevented the reliable collection of data for these samples at 30 wt%. In contrast, the 3D structure of graphite allowed for better dispersion within the polymer matrix, maintaining the integrity of the sample even at 30 wt%. Therefore, the results for CFs at 30 wt% were not included in Figure 11. The shape fixation ratio of CFs varied with length, with shorter CFs showing a higher shape fixation ratio. The shorter-length CFs were uniformly mixed within the polymer matrix and had less recovery from a fixed shape to the original shape. As a result, the shorter-length CFs showed a higher shape fixation ratio.

**Figure 12:**
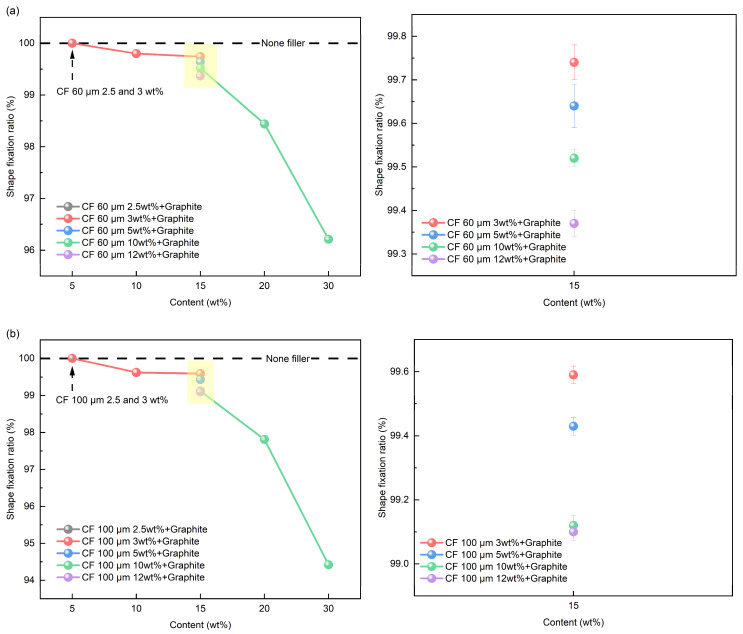
Shape fixation ratio results of mixed filler: (**a**) 60 μm CF; (**b**) 100 μm CF.

The shape fixation ratio of SMP with mixed filler was analyzed using the contents in Table 2. For the 15 wt% mixed filler, different ratios of graphite and CF were used (3:12, 5:10, 10:5, 12:3). Figure 12 shows the shape fixation ratio results of the SMP with mixed fillers, where Figure 12a,b show the results with 60 and 100 μm CFs, respectively. In Figure 12a the data point of CF 60 μm 2.5 wt% + Graphite at 5 wt% overlap with the data of CF 60 μm 3 wt% + Graphite. The data of CF 100 μm 2.5 wt% + Graphite and CF 100 μm 3 wt% + Graphite also have same fixation ratio at at 5 wt% in Figure 12b. The use of mixed fillers decreased the shape fixation ratio with increasing content. However, the mixed filler showed a higher shape fixation ratio at the same content. In particular, a 100% shape fixation ratio was achieved at 5 wt%. Also, no fractures occurred during the mold fixation process at 30 wt%. The 3D structure of the mixed filler effectively limits the movement of the polymer chains and is advantageous for the conservation of strain energy required for shape recovery [27]. When comparing the fixation ratio at 15 wt% with different contents of graphite and CF, the higher graphite content improved the shape fixation ratio. The flexibility and toughness of graphite are thought to have had a positive effect on the shape fixation ratio. The results show that using mixed filler with high graphite content improves the shape fixation ratio of the SMP. The shorter the length of the CF, the higher the shape fixation ratio becomes. Shorter CF, which possesses lower stiffness compared to longer CF, is more advantageous for shape fixation. Higher stiffness increases resistance to mechanical stress, making shape fixation more challenging. Generally, a shape fixation ratio of 90% or more is considered to indicate successful shape recovery performance. Notably, SMP with mixed filler demonstrated superior shape recovery performance in comparison to SMP with a single filler at all filler contents.

#### 3.3.2. Shape Recovery Ratio

**Figure 13:**
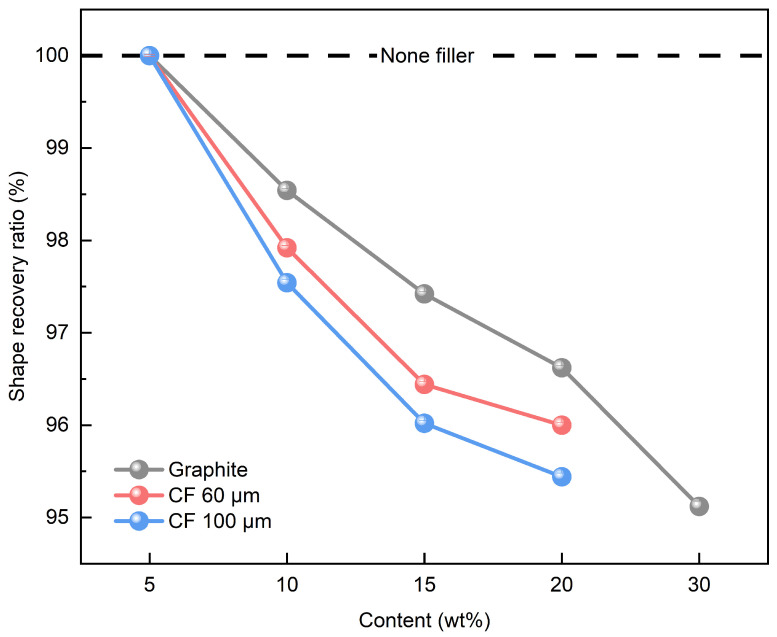
Shape recovery ratio results of single filler.

As shown in Figure 4, SMP recovery is the process in which a shape, once fixed below T_g_, returns to its original form when it is above T_g_. Above T_g_, the polymer chain releases stored strain energy, as a result of the entropy change-induced elasticity. The shape recovery ratio of the SMP without filler was measured to be 100%. The shape recovery ratio of SMP with a single filler was analyzed using the contents in Table 1. Figure 13 shows the shape recovery ratio of SMP with a single filler. Similar to the shape fixation ratio findings, the shape recovery ratio decreased as the filler content increased, with graphite exhibiting a higher shape recovery ratio compared to CF. As in the results shown in Figure 11, CFs excluding graphite experienced fracture at 30 wt%, and therefore, were excluded from Figure 13. Shorter CF lengths were associated with higher shape recovery ratios. Additionally, when the filler content was 5 wt%, the shape recovery ratio reached 100%, unlike the shape fixation ratio. This could be because, as explained in the shape fixation ratio, the 2D structure of graphite effectively preserves the deformation energy required for shape recovery. Similarly, uniformly mixed short CFs in a polymer matrix appear to be more effective at preserving deformation energy compared to long CFs.

**Figure 14:**
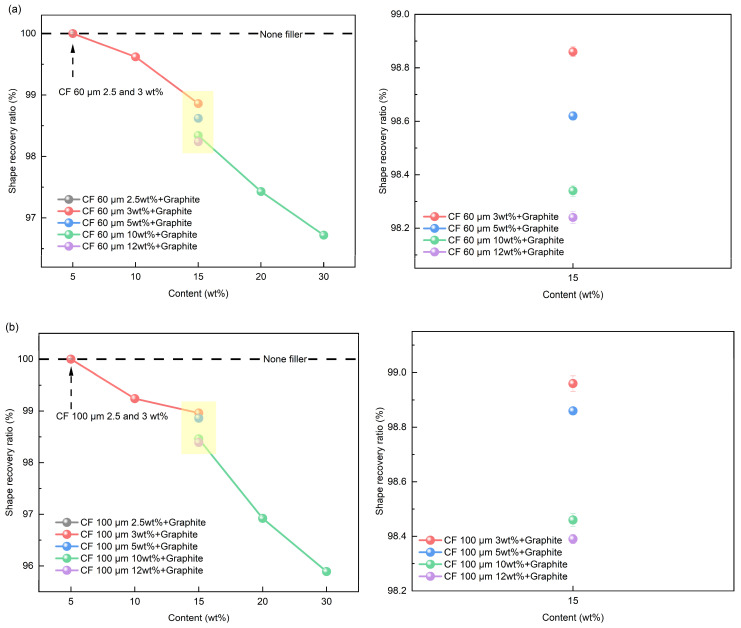
Shape recovery ratio results of mixed filler: (**a**) 60 μm CF; (**b**) 100 μm CF.

The shape recovery ratio of SMP with mixed filler was analyzed using the contents in Table 2. For the 15 wt% mixed filler, different ratios of graphite and CF were used (3:12, 5:10, 10:5, 12:3). Figure 14 shows the shape recovery ratio results of the SMP with mixed fillers, where Figure 14a,b show the results with 60 and 100 μm CFs, respectively. For the initial content of 5wt%, all samples shows 100% of shape recovery ratio in Figure 14a,b. Although the shape recovery ratio tends to decrease as filler content increases, similar to the single filler, the mixed filler exhibits a superior shape recovery ratio at the same content. This enhancement can be attributed to the 3D structure formed by the graphite and CF bonding, effectively retaining strain energy compared to the 1D or 2D structures. This stored energy is released during the shape recovery process, resulting in an improved shape recovery ratio [27]. When comparing the shape recovery ratios while varying the graphite and CF content, the highest shape recovery ratio was observed with a graphite content of 15 wt%. Furthermore, just as with the shape fixation ratio, the use of mixed fillers yielded a better shape recovery ratio when the graphite content was high, and the CF length was short. As with the shape fixation ratio, it can be concluded that the shape recovery ratio meets the criterion for shape recovery performance when it exceeds 90%. Thus, it was determined that both single and mixed fillers in SMP satisfy the shape recovery ratio criterion, with the mixed fillers providing a better shape recovery ratio.

#### 3.3.3. Shape Recovery Time

**Figure 15:**
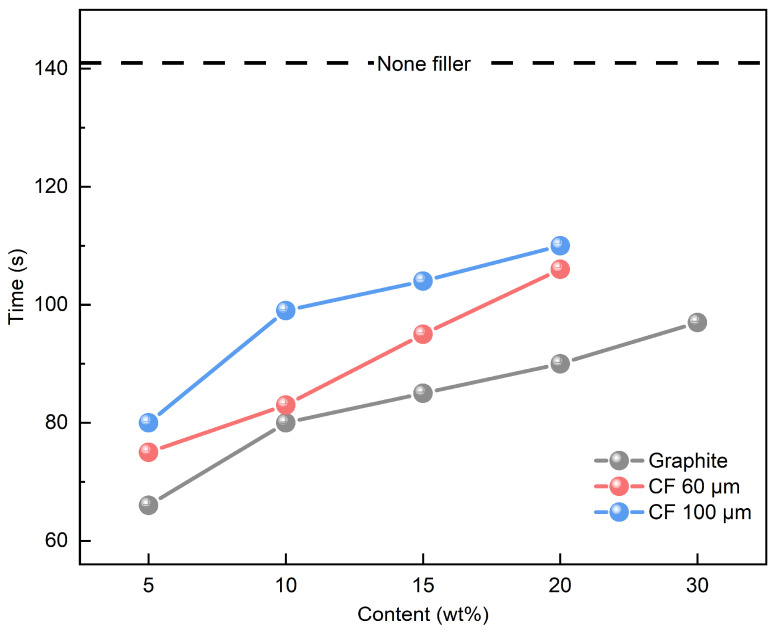
Shape recovery time results of single filler.

The shape recovery time is defined as the duration required for the SMP to return from its deformed state to its original shape. It took 141 s for the SMP to recover its original shape. In the case of an SMP containing a single filler, Figure 15 shows the shape recovery time based on Table 1. Notably, despite the enhanced thermal conductivity resulting from filler incorporation (0.2296 W/mK for a single SMP), an increase in filler content led to a prolonged shape recovery time. As elucidated in Figure 8 and Figure 9, the predominant interaction forces between the fillers within the polymer matrix were primarily van der Waals forces, and these forces intensified with rising filler content. Importantly, these forces surpassed the deformation energy required for shape recovery, exerting a detrimental influence on the shape recovery time. It was observed that graphite, possessing the highest thermal conductivity, exhibited a shorter shape recovery time compared to CF. At a graphite content of 5 wt%, the time required was reduced to 66 s, representing a significant 53.19% reduction. Furthermore, as depicted in the DSC results in Figure 7, the reduction in the T_g_ attributed to filler utilization contributed to the abbreviated shape recovery time of the SMP. This decrease in T_g_ had a favorable impact on augmenting ∆T within the operational temperature range of the SMP, which was set at 100 °C.

**Figure 16:**
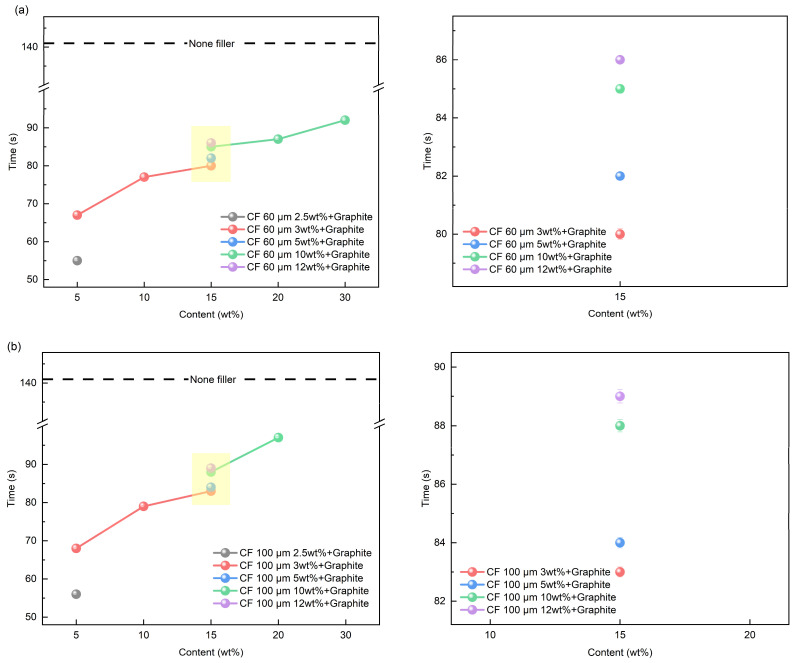
Shape recovery time results of mixed filler: (**a**) 60 μm CF; (**b**) 100 μm CF.

Using the content ratios outlined in Table 2, Figure 16 shows the shape recovery time of SMP employing mixed fillers. For a 15 wt% mixed filler composition, graphite and CF were blended in different ratios (3:12, 5:10, 10:5, 12:3). Figure 16 portrays the shape recovery time of SMPs with mixed fillers, with Figure 16a depicting the sample incorporating 60 μm CF and Figure 16b showcasing the sample featuring 100 μm CF. It becomes evident that as the proportion of mixed filler increases, the shape recovery time becomes more extended. This trend aligns with the findings observed with single fillers, but notably, it results in a swifter shape recovery time when compared to an equivalent quantity of a single filler. This enhancement is attributed to the superior capacity for strain energy storage facilitated by the 3D structure inherent in mixed fillers. Additionally, the lower T_g_ contributes to a greater variance in ∆T concerning operating temperatures, which is believed to positively impact the shape recovery time. We also conducted a comparison of shape recovery times at 15 wt% while varying the graphite and CF content. As demonstrated in Figure 10, the heightened graphite content enhances thermal conductivity, thereby promoting a faster shape recovery time. Furthermore, the variations in shape recovery time concerning CF length followed a pattern akin to that observed for shape fixation and shape recovery ratios. In summary, it is evident that the incorporation of mixed fillers consistently improves the shape recovery time of SMP, particularly when accompanied by high graphite content, especially when combined with short CF lengths.

## 4. Conclusions

Various carbon-based fillers with different shapes and proportions were employed to enhance both thermal properties and shape recovery in SMP. The incorporation of 3D structured fillers in the blending process emerged as a pivotal factor in enhancing the overall characteristics of the SMP. These 3D structures established efficient pathways for heat transfer, proving advantageous for the efficient storage and release of strain energy. Notably, an increase in the graphite content, a 2D structured filler within the same composition, not only improved thermal properties but also significantly enhanced shape recovery performance. When mixed fillers were utilized, there was a remarkable 290.37% increase in thermal conductivity for SMPCs containing 60 μm CF 10 wt% + graphite 20 wt% compared to single fillers, underscoring the exceptional shape fixation and recovery capabilities. Furthermore, the shape recovery time was substantially reduced by up to 60.99% for SMPC containing 60 μm CF 2.5 wt% + graphite 2.5 wt%. Particularly noteworthy was the performance of the 15 wt% SMP with higher graphite content compared to CF. In this scenario, thermal conductivity improved by 37.42%, while shape recovery time was shortened by 6.98%. Utilizing higher graphite content relative to CF in the mixture emerged as a successful strategy for enhancing thermal properties and shape recovery performance, thereby demonstrating the applicability of SMP in situations requiring rapid responses.

## Figures and Tables

**Table 1:** Weight ratio of single fillers.

Single Filler	
Filler Type	Content (wt%)
60 μm CF	5, 10, 15, 20, 30
100 μm CF	
Graphite	

**Table 2:** Weight ratio of mixed fillers.

Mixed Filler		
Filler Type		Content (wt%)
60, 100 μm CF	Graphite	
2.5	2.5	5
3	2	
3	7	10
3	12	15
5	10	
10	5	
12	3	
10	10	20
10	20	30

**Table 3:** Changes in T_g_ according to the epoxy resin to hardener ratio.

Epoxy Resin/Hardener	T_g_ (°C)
8:1	83.60
9:1	75.77
10:1	69.41

## Data Availability

Data are contained within the article.

## References

[1] Xue J., Ge Y., Liu Z., Liu Z., Jiang J., Li G. (2022). Photoprogrammable moisture-responsive actuation of a shape memory polymer film. ACS Appl. Mater. Interfaces.

[2] Wang Y., Wang Y., Wei Q., Zhang J. (2022). Light-responsive shape memory polymer composites. Eur. Polym. J..

[3] Xu Z., Ding C., Wei D.W., Bao R.Y., Ke K., Liu Z., Yang W. (2019). Electro and light-active actuators based on reversible shape-memory polymer composites with segregated conductive networks. ACS Appl. Mater. Interfaces.

[4] Sachyani Keneth E., Lieberman R., Rednor M., Scalet G., Auricchio F., Magdassi S. (2020). Multi-material 3D printed shape memory polymer with tunable melting and glass transition temperature activated by heat or light. Polymers.

[5] Melly S.K., Liu L., Liu Y., Leng J. (2020). Active composites based on shape memory polymers: Overview, fabrication methods, applications, and future prospects. J. Mater. Sci..

[6] Chen Y., Chen C., Rehman H.U., Zheng X., Li H., Liu H., Hedenqvist M.S. (2020). Shape-memory polymeric artificial muscles: Mechanisms, applications and challenges. Molecules.

[7] Bhanushali H., Amrutkar S., Mestry S., Mhaske S.T. (2022). Shape memory polymer nanocomposite: A review on structure–property relationship. Polym. Bull..

[8] Kim M., Jang S., Choi S., Yang J., Kim J., Choi D. (2021). Analysis of shape memory behavior and mechanical properties of shape memory polymer composites using thermal conductive fillers. Micromachines.

[9] d’Almeida J.R.M., Monterio S.N. (1993). The effect of the resin/hardener ratio on the compressive behavior of an epoxy system. Polym. Test..

[10] d’Almeida J.R., Monterio S.N. (1998). The influence of the amount of hardener on the tensile mechanical behavior of an epoxy system. Polym. Adv. Technol..

[11] Lendlein A., Gould O.E. (2019). Reprogrammable recovery and actuation behaviour of shape-memory polymers. Nat. Rev. Mater..

[12] Chen L., Li W., Liu Y., Leng J. (2016). Nanocomposites of epoxy-based shape memory polymer and thermally reduced graphite oxide: Mechanical, thermal and shape memory characterizations. Compos. B Eng..

[13] Wang E., Dong Y., Islam M.Z., Yu L., Liu F., Chen S., Hu N. (2019). Effect of graphene oxide-carbon nanotube hybrid filler on the mechanical property and thermal response speed of shape memory epoxy composites. Compos. Sci. Technol..

[14] Li M., Ali Z., Wei X., Li L., Song G., Hou X., Yu J. (2021). Stress induced carbon fiber orientation for enhanced thermal conductivity of epoxy composites. Compos. B Eng..

[15] Wei J., Liao M., Ma A., Chen Y., Duan Z., Hou X., Yu J. (2020). Enhanced thermal conductivity of polydimethylsiloxane composites with carbon fiber. Compos. Commun..

[16] Bao D., Gao Y., Cui Y., Xu F., Shen X., Geng H., Wang H. (2022). A novel modified expanded graphite/epoxy 3D composite with ultrahigh thermal conductivity. Chem. Eng. J..

[17] Wang Z., Qi R., Wang J., Qi S. (2015). Thermal conductivity improvement of epoxy composite filled with expanded graphite. Ceram. Int..

[18] Wu K., Lei C., Huang R., Yang W., Chai S., Geng C., Fu Q. (2017). Design and preparation of a unique segregated double network with excellent thermal conductive property. ACS Appl. Mater. Interfaces.

[19] Zhang F., Feng Y., Feng W. (2020). Three-dimensional interconnected networks for thermally conductive polymer composites: Design, preparation, properties, and mechanisms. Mater. Sci. Eng. R Rep..

[20] Jasmee S., Omar G., Othaman S.S.C., Masripan N.A., Hamid H.A. (2021). Interface thermal resistance and thermal conductivity of polymer composites at different types, shapes, and sizes of fillers: A review. Polym. Compos..

[21] Lu H., Yu K., Sun S., Liu Y., Leng J. (2010). Mechanical and shape-memory behavior of shape-memory polymer composites with hybrid fillers. Polym. Int..

[22] Sun Y., Zhang Z., Moon K.S., Wong C.P. (2004). Glass transition and relaxation behavior of epoxy nanocomposites. J. Polym. Sci. B Polym. Phys..

[23] Zhang D., Huang Y., Chia L. (2022). Effects of carbon nanotube (CNT) geometries on the dispersion characterizations and adhesion properties of CNT reinforced epoxy composites. Compos. Struct..

[24] Zhang D., Huang Y., Xia W., Xu L., Wang X. (2024). Dispersion characteristics and mechanical properties of epoxy nanocomposites reinforced with carboxymethyl cellulose functionalized nanodiamond, carbon nanotube, and graphene. Polym. Compos..

[25] Shin H., Yang S., Choi J., Chang S., Cho M. (2015). Effect of interphase percolation on mechanical behavior of nanoparticle-reinforced polymer nanocomposite with filler agglomeration: A multiscale approach. Chem. Phys. Lett..

[26] Ervina J., Mariatti M., Hamdan S. (2016). Effect of filler loading on the tensile properties of multi-walled carbon nanotube and graphene nanopowder filled epoxy composites. Procedia Chem..

[27] Dusoe K.J., Ye X., Kisslinger K., Stein A., Lee S.W., Nam C.Y. (2017). Ultrahigh elastic strain energy storage in metal-oxide-infiltrated patterned hybrid polymer nanocomposites. Nano Lett..

